# Religious Affiliation, Daily Spirituals, and Private Religious Factors Promote Marital Commitment Among Married Couples: Does Religiosity Help People Amid the COVID-19 Crisis?

**DOI:** 10.3389/fpsyg.2021.657400

**Published:** 2021-08-06

**Authors:** Jaffar Aman, Jaffar Abbas, Umi Lela, Guoqing Shi

**Affiliations:** ^1^Postdoctoral Station, School of Public Administration and Sociology, Hohai University Nanjing, Nanjing, China; ^2^Antai College of Economics and Management (ACEM), School of Media and Communication (SMC), Shanghai Jiao Tong University (SJTU), Shanghai, China; ^3^Head of Department, Humanities and Social Sciences, Gift University, Gujranwala, Pakistan; ^4^School of Public Administration, Hohai University Nanjing, Nanjing, China

**Keywords:** marital commitment, religiosity, COVID-19, emotional attachment, age difference, marriage terms

## Abstract

Religious studies are a vital branch of social science that seeks to explain the beliefs of human society and deals with the practices and beliefs of individuals. This distinctive study focuses on such influential aspects of a healthy life, which could play a vital role in the marital quality and matrimonial commitment of individuals. The study principally focused on inspecting the role of religiosity in healthy marital commitment among individuals. It is a distinctive and central value in regulating a healthy social life. This research designed a conceptual model for assessing marital commitment, and the study model comprised two primary variables. The study received datasets through a survey questionnaire based on participants from five private and public sectors. The research study conducted an empirical analysis to test the proposed conceptual framework. The findings exhibited that the value of the *R*^2^ model was 0.484, meaning the level of religiosity had a substantial impression on healthy and lasting marital commitment. According to the final outline of the model factors associated with building religious support factors (β = 0.491), the marital commitment had a better and healthier impact. The goodness-of-fit of the measurement of the conceptual model showed a value of 0.51, which indicated that the theoretical model had sufficient consistency and rationality, and accurately fitted the data. Such an advanced statistical model is missing from the previous literature. The study results provide helpful insight to elucidate the social dynamics of marital commitment. The findings designate that religious practices strengthen and promote nuptial commitment. The study is novel in the context of religiosity impact on martial commitment with a cultural background of Pakistan. The generalizability of the study does not apply to the entire population or other regions. Future studies can investigate other religious variables to explore further research findings. The findings are helpful for decision-makers and policymakers to concentrate on marital issues and challenges confronted by couples worldwide.

## Introduction

Several researchers have recently assessed the connection between religion and marital commitment in many areas of family studies. In general, previous studies have shown that people with the highest level of religiosity had lower levels of marital commitment and an improved quality of life in modern societies, as well as a global cultural influence; and these factors are essential for people, as they have social impacts that directly affect daily lives of people. Communities with better living conditions have improved living standards and social welfare among married women (Bredemeier et al., 2012). In recent times, individuals have preferred to live in better communities and have preferred the best partners to ensure happy and peaceful lives. In a previous study, Zahra Alghafli has explored Islamic teachings, family relationships, and marital satisfaction from a domestic perspective of couples. The study also examined how married respondents identified and described critical problems; for instance, the fundamental rights of women, family unity, and gender responsibilities. Therefore, it is vital to understand how marital commitment or marital satisfaction develops and how it contributes to increasing marital satisfaction and managing marital relationships in different ways (Alghafli et al., 2014). Marriage is a traditional social and ceremonial association of partners with recognized duties and rights. The standard description of marriage differs by culture and region, since marriage is recommended as a religious obligation. As a result, marriage is an agreement between a couple to show love, commitment, and pleasure, and to commit to a healthy family relationship. This is the foundation of better mental fitness in women (Van der Borght et al., 2016). Therefore, emotional and legal consent in the life of every married man and woman is critical. In addition, the choice of spouse and the marriage contract are considered to be personal benefits in adulthood. Choosing a spouse is indeed one of the most difficult decisions in one's life (Liu et al., 2017). Individuals get married for various reasons, such as pleasure, love, aspiration to create a family, sexual need, or desire to escape isolation and societal hardship (Ozguc and Tanriverdi, 2018).

Thus, families will extend their lives to incorporate a profession while retaining their collective share. They will improve their lives to consolidate a calling, while at the same time keeping their regular parts. After completing her office work, a mother comes home to take care of her children and partner. Her office and home duties make up an exceptionally complex plan (Ritvo and Glick, 2005; Aziz and Cunningham, 2008). Therefore, the importance of family for couples is very significant, which makes a marriage stable. It should also be noted that family and marital relationships remain one of the most discussed issues in the modern world (Ritvo and Glick, 2005). Thus, the most critical connection between both men and women is marriage, which guarantees a stable family life. It includes a passionate and appropriate duty, which is essential for an adult. According to Bernard, people prefer to marry to enjoy life experiences with their spouses. They can feel the spirit of love, friendship, ecstasy, the blessing of children, the physical desire to please, and can escape a sad situation (Cook and Dickens, 2009). The term “religiosity” is not clearly defined, as various scholars consider it in a broad sense as related to religious obligations and tendencies. Religiosity represents and exhibits multiple factors, such as experience, ritual, ideologies, coherence, rationality, belief systems or faith, morality, and cultural dimensions. Sociologists describe religion as a feeling in people, a sense of justice, and the behavior of people who generally disagree with their actual religious beliefs. There are different definitions, according to which you can truly practice your religion and feel assured or at ease (Langlais and Schwanz, 2017).

This study emphasized examining religious affiliation, practices, and spiritual attitudes of individuals and how these elements affect marital commitment among couples. The study investigated the association between religious participation, practices, and marital quality of individuals to determine its likely impact on the marital commitment of individuals. This study aimed to address this question:

What is the possible linkage between religious experience, religious affiliation, and marital commitment of people? Past literature documented some studies conducted in the United States and other regions, and these research studies primarily focused on exploring the relationship between religiosity elements and marital commitment. This study has identified this literature gap in the context of Pakistan, and it is the first research model to explore these factors. The study focuses on how these elements of religiosity influence the quality of marital commitment among people. This article explores how religiosity, such as religious beliefs, religious practices, and religious commitment, affects the quality of marital commitment of Pakistani married couples. This research model intends to contribute to the existing literature in the domain of marital commitment and its association with religiosity factors. The study investigates how these religious elements influence marital quality among married people in Pakistan.

### Literature Review

After several years of study by renowned scientists, such as sociologists, psychologists, and anthropologists, a religious census has been established that hypothesizes that religious beliefs of people are firmly embedded in their minds. These religious concepts are fragmented, associated with the insecurity and dependency of the situation and method, as if other identical areas are based on culture and life Beliefs, behaviors, relationships, and associations of people are diverse activities that are related to other factors, such as culture. Marital commitment and satisfaction are unique cases in creating a satisfactory level for partners to evaluate their marital relationship from various aspects of the life of an individual. In general, the value of marital commitment shows the degree of pleasure and gratification of people in their marital affiliation (Zaloudek, 2014). Therefore, this study found a connection with other influential or sad life events, such as the transition to parenthood, stressful work atmosphere, economic stress, and poor health of one's spouse, factors associated with low marital quality and infertility and its treatment. In contrast, couples with low-quality marriages are exposed to health risks that have psychological consequences, such as anxiety, stress, and depression, which cause health problems.

On the contrary, the family is the primary base of human society. It creates a workforce for future generations and other social organizations, since familiarity or deviation depends on general circumstances of the family structure, as social losses cannot occur without the influence of the family. Thus, several factors influence the firm and consistent definition of family, but marital satisfaction is critical to the mental health of couples and children. Marriage is always the basis of social support for people and is seen as protecting against the devastating consequences of life, such as mental health problems and other undesirable events. According to Hawkins and Booth (2005), unhappily married people received a significantly higher rating on their distress evaluation (Hawkins and Booth, 2005). They were not satisfied with their married life and had lower scores for life gratification compared with couples who were relishing a happier and joyful married life. However, married couples with troubled lives face health risks, such as physical and mental suffering. As a result, it is important to identify factors that can slow the decline in marital satisfaction among people, and religion and spirituality are factors associated with marital satisfaction. Family is the oldest social institution, which has endured in society since the birth of humankind. It begins with marriage, because the stability or immaturity of the marriage determines the durability of the relationship (Fard et al., 2013). Several factors influence marital commitment and trust. Intimacy is one of the essential elements necessary to strengthen a marital relationship, creating more love and avoiding separation. Therefore, two of the most critical human needs are a reliable emotional connection and a secure attachment with a life partner, which influence the lives of married couples and family connections (Krystosek, 2016). Attachment theory relates to relationships between adults (Juffer et al., 2017). An earlier study (Kogut, 2016) stated that adults who avoid attachment consider themselves useless to others.

These individuals refute their susceptibility to attachment and argue that they do not need intimate relationships; they try not to become close to other people in society. Commitment is seen as a choice to continue the family life and is related to a mental connection with a life partner (Yamaguchi et al., 2017). A study (Deniz and Yozgat, 2013) argued that marriage commitment refers to how much a couple considers the values of their marriage and how much they want to maintain and continue their family life. Commitment provides a stable foundation for strengthening their relationship and can act as a facilitator for forgiveness and sharing of mutual feelings (Back, 2010). Commitment is, therefore, a significant element in maintaining a spousal relationship (Komura, 2014).

Marriage plays a vital role in sustaining a romantic marital relationship. It has always been seen as the first emotive commitment and legal right that adults recognize. It creates a strong relationship between couples and offers the highest level of social responsibility, helping couples experience, emotions and it helps meet their need for safety (Uysal, 2016). Marriage can be seen as one of the most fundamental choices for every person in their youth. Previous studies have shown that loyal people with similar religious beliefs and attachments are known as homogeneous couples. They are married couples who are generally satisfied with the quality of their marriage. Marital commitment for couples is, therefore, one of the most delicate issues in their lives. It is an essential concept in family life, as it has been the subject of numerous previous studies (Caki et al., 2015; Perry et al., 2016). In terms of emotional attachment, marriage is proof of commitment and a promise of emotional attachment (Inoue et al., 2017). Marital commitment is the level at which people have a long-standing perception of marrying and sacrificing their relationships to maintain and strengthen their solidarity and unity with their spouse, even if their marriage is not rewarded (Stowell, 2018). Consequently, commitment is divided into three different parts: ethical commitment, fundamental commitment, and personal commitment.

The three components combine to create a satisfying relationship between people. Personal commitment refers to the interest of a person in continuing their marital relationship based on marital gravity and marital satisfaction. Ethical obligation denotes the responsibility that people believe they have to stay connected and enjoy life. Structural commitment implies the existence of social relationships. Some authors believe that religious values are predictors of marital commitment (Murray and Holmes, 2015; Tseng et al., 2017). Religion seems to be a crucial factor in strengthening marital responsibility. Religion is a combination of beliefs and practices supported by religious institutions. Religiousness is defined in terms of beliefs and behaviors of individuals in maintaining relationships with spiritual values and convincing values. As a result, people with religiosity mention their religious beliefs and practices, and express feelings that other people do not usually see (Green and Douglas, 2018). The factors that indicate individual religiosity include thoughts, feelings, devotion to God, worship, and spiritual inquiry (Liu and Hao, 2017). Universal religiosity involves participating in religious rites and group worship, and being present in places of worship (Evensen and Stedman, 2017).

Marital commitment and marital satisfaction are crucial for people who were happy and satisfied with their lives before choosing their partner. Several well-known scientists have examined and presented detailed literature on the effects of religiosity on marriage commitment. The researchers discussed and identified how religiosity is one of the factors that influence satisfaction of individuals with marriage, because religiosity controls and determines the structure of their religious beliefs, norms, values, practices, and ideas. It transmits a lifestyle that can have an impact on the quality of marital life (Lichter and Carmalt, 2009). In due time, religion controls and determines beliefs of people about household chores; the way they love their partner, and how they ensure peace, loyalty, and reality of their family. This demonstrates the influence of religion on better marital relationships. The Holy Book of the Quran calls marriage a social bond among men and women, a firm contract, since both sides enjoy comfort and relaxation because of marital gratification. Religion always emphasizes divine relationships among people, and previous studies have shown that religious beliefs of married women are correlated with marital satisfaction (Juvan and Dolnicar, 2017). Ultimately, shared expectations of partners can offset the impact of economic pressure and protect people from financial stress and its harmful effects on the health of individuals (Pulles and Hartman, 2017).

In prior research studies, scholars argued that divorce rates would be lower if couples have more inclination toward religion (Aman et al., 2019). Religion enables marriages to endure and is useful in some other situations, as their results confirmed that parents with greater religiosity had a substantial effect on the adaptation of their children (Mahoney et al., 2001). In another research study, Marx interviewed 76 very religious married couples from Muslim, Jewish, Mormon, and Christian communities to analyze the impact of three aspects of religion (spiritual practice, religious belief, and faith) on marriage. His findings showed that religiosity, reliance on God, and religious views improved the performance of marriage between faith-minded couples (Marks, 2005). In their research study, David and Stafford (2013) examined the relationship between religion and spirituality. They explored the relationship of people with God, mutual interactions, and forgiving behavior by religious couples as predictors of marital gratification. The study revealed that a spiritual connection with God is a critical factor in determining the quality of a spousal relation, as married couples seemed to express themselves through religious interactions, which were directly related to the quality of their marriage (David and Stafford, 2013).

In the history of the human race, regardless of the religious views involved, there are signs of worship. Studying the course of human civilization also shows that with the development of human knowledge and attention to the metamorphic world, growth can be observed (Jones and Heley, 2016). Since both institutions emphasize similar values of religion and family and are interdependent when it comes to improving socialization, the researchers anticipate a close relationship between them. Scholars have argued that religiosity can strengthen marital bonds (Gholipour and Farzanegan, 2015). Religious manners can be beneficial to marital relationships, because religion provides guidelines for life, and the beliefs and values of an organization are reflected in family life. Faith in God makes the attitude of a person more general and meaningful. Trust in God breaks cohesion. The human world demoralizes relationships and is the source of dissimilarities (Markham, 2012). Couples who are drawn to the *masjid* (mosque) or church and perform religious obligations, such as religious rituals and pilgrimages, typically show a lower rate of divorce and enjoy a stable married life (Carmichael, 2011). The features of healthy families are different, but the relationship between religion and family participation is systematically clear (Agha, 2016). In his research, he also found that people of religious origin, devotion, and satisfaction had better marriages than those without a religious connection. Given the importance of participation in married life to maintain the mental health of couples and society as a whole, there is a need for actions to improve family relationships and, in particular, to improve marital relationships for couples.

However, to realize this, we must first find relevant information about this structure and determine the factors that influence it. Couples must follow the appropriate steps to create and maintain a marriage. Considering that religiosity is one of the most influential factors in marital satisfaction, happy marriages lead to durable marriages and to spouses staying married, and make family relationships secure. Conversely, a non-committed marital life can lead to infidelity and, ultimately, divorce and separation. Previous studies debated marital commitment and explained it closely related to marital satisfaction. It has a connection with religiosity. This study also inspected the role of religion and its aspects in predicting conjugal commitment among married couples living in five different regions of Pakistan.

In assessing useful religious aspects that impact marital commitment among Muslim individuals, this study used the SEM (structural equation modeling) technique to test the conceptual design model. Thus, considering the objective of this study, the researcher used the theory of commitment to give the research direction. Commitment is a fundamental notion used to understand the continuation of human relationships in Pakistani society. Behavioral and social scientists have also developed various theories on the marital commitment of people in the past several years. George Levinger conducted studies, introduced the commitment theory, and mainly focused on understanding and separating the processes involved in maintaining relationships (specifically marriages). See Tables 1, 2 for further details.

**Table 1:** Data sources and methodologies of previous studies.

**Sr.**	**References**	**Title**	**Method**	**Country**
1	DeLongis and Zwicker (2017)	Marital Satisfaction and Divorce in Couples in Stepfamilies (*N* = 112)		Canada
2	Khalatbari et al. (2013)	The Relationship between Marital Satisfaction (Based on Religious Criteria) and Emotional Stability (*N* = 110)	Correlation analysis	USA
3	Van Mol and de Valk (2016)	Relationship Satisfaction of European Binational Couples in the Netherlands (*N* = 285)	Regression analysis	Netherlands
4	Madanian et al. (2013)	Marital Satisfaction of Iranian Female Students in Malaysia: A Qualitative Study (*N* = 10)	Inductive thematic analysis	Malaysia
5	Chung (2014)	Pathways between Attachment and Marital Satisfaction: The Mediating Roles of Rumination, Empathy, and Forgiveness (*N* = 208)	Structural equation modeling	Korea
6	Karukivi et al. (2014)	Is Alexithymia Linked with Marital Satisfaction or Attachment to the Partner? A Study in a Pregnancy Cohort of Parents-to-be (*N* = 112)	Spearman correlation analyses	Finland
7	Pilar Matud et al. (2014)	The Relevance of Gender Roles in Life Satisfaction in Adult People (*N* = 1,233)	Pearson's correlation coefficient	Spain

*The data sources and methodologies of previous studies focusing on marital satisfaction. Primary factors list*.

**Table 2:** Scale items used to measure the study constructs.

**Sr. no.**	**Scale items**
1	I can feel God/Allah's presence
2	During worship or other times, I feel joy that lifts me out when connect with God
3	I can feel strength in my spiritually or religion
4	I usually feel comforts/strength in my spiritually or religion
5	I can directly feel love of God for me
6	I desire to get closer with God or try to in union with my Lord
7	I often participate in religious services/activities
8	I love in paying attention to my daily religious activities/services
9	I do believe in worship
10	My family does not force me to participate in daily spiritual/religious activities
11	I feel that people in my congregation make me feel loved and cared for
12	I feel that people in my congregation listen to me and talk about my concerns and problems
13	I feel that people in my congregation express interest to my concerns for personal well-being
14	I feel that my husband is honest and truthful person with me
15	I feel that my husband is fully trustable partner for my life.
16	I feel that my husband is sincere and trustworthy in his promises with me
17	I observe that my husband typically treats me fairly
18	I feel that my husband is trustworthy and helps me when I need it

*This Table shows the influencing factors of the study*.

## Conceptual Model

Scholars widely employ the partial least squares-structural equation modeling (PLS-SEM) tool, which is a broader multivariable evaluation method for calculating the structural equation's variance-based models, particularly in social science (Sarstedt et al., 2014; Rönkkö et al., 2016). Scholars widely apply the SEM technique to examine multifaceted connotation systems and pivotal interactions, such as route models for hidden variables and their perceived exponents (Sarstedt et al., 2014; Schubring et al., 2016). The SEM approach is a combination of regression, complex correlation, factor evaluation, and path assessment. The theoretical model clarifies the contacts between the latent and corresponding observable variables. The primary stage focuses on identifying the latent variables in the organizational model and forms hypotheses for evaluation, such as on satisfaction and quality. Since the latent (unobservable) variables are not measured directly, the model has to regulate these variables (observable) under study.

**Figure 1:**
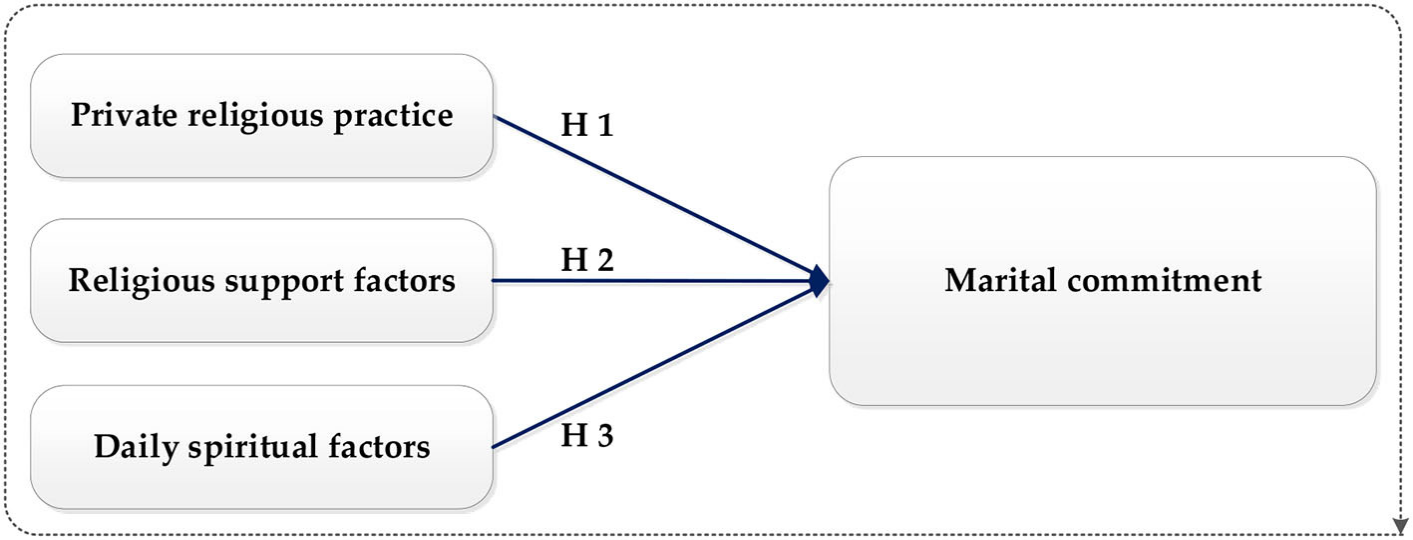
Conceptual framework of marital commitment.

This study developed a model based on a combination of 13 visible variables that affect marital commitment. These factors determine the exogenous latent variables, consisting of three factions: factors associated with daily spirituality, religious support, and private religious practices. The study measured the chosen variables directly using a five-point Likert scale. Figure 1 explains the conceptual and theoretical model, representing the relationship between the explicit exogenous, explicit endogenous, and latent variables. The term “latent dependent variables” refers to the endogenous or dependent variables (DVs), which specify that the independent latent variables (IVs) affect the endogenous variables (EVs) (Krajangsri and Pongpeng, 2017), see Figure 1.

### Research Hypotheses

Hypothesis 1: Private religious practices have a strong positive influence on marital commitment.
Hypothesis 2: Daily spiritual factors have a significant relationship with marital commitment.
Hypothesis 3: Religious support factors have a positive relationship with marital commitment.

## Methodology

Scholars characteristically apply statistical tools, such as smart-PLS-SEM and SPSS, to develop research theories in exploratory research studies (Fursova, 2018). The smart-PLS-SEM tool refers to vital applications based on confirmatory factor analysis (CFA), path analyses, various regression based models, second-order factor analyses, correlation structure models, and covariance structure models (Hall et al., 2008). The SEM approach is useful for examining linear associations between latent construct(s) and manifest variable(s). Hence, the partial least squares method describes a multivariate statistical process to evaluate a measurement model instantaneously. It examines the relationships between constructs of the designed model and related conforming study indicators through the structural model to indicate a linkage between the constructs (Hair et al., 2013). The SEM technique helps in estimating parameters to evaluate the relationships between unobserved variables. Thus, SEM approaches help permit many relationships to examine, calculate, and test at once in a single projected model with several connotations instead of testing every single connection independently. This proposed research tested the hypothesized framework, as indicated in **Figure 3**, and incorporated the PLS-SEM method to assess latent constructs of the selected model using numerous manifest variables. This method is helpful compared with other regression-based approaches in measuring variable relationships (Hall et al., 2008). The PLS-SEM research technique is a better, flexible, and robust method for building a suitable and computable statistical framework/model, and it helps to accomplish predictive study objectives (Lowry and Gaskin, 2014; Wise, 2016; Hair et al., 2017). Many scholars explained that valid, trustworthy (Hair et al., 2020), and reliable CFA is computable through a PLS-SEM path modeling approach (Astrachan et al., 2014). The smart PLS is a consistent and reliable tool that helps various researchers of business and social sciences to test proposed models to draw results (Hair et al., 2017). This tool can easily handle abnormal statistical datasets because of its flexible assumptions related to variable distribution and normality (Henseler et al., 2009). Besides, this method permits researchers to test complex proposed research models based on multi-levels, such as moderating, mediating, and other complex connections of selected study variables (Lowry and Gaskin, 2014). This research has employed the latest version of PLS-SEM 3.3.3 for analysis purposes and measured path coefficients, weights, loadings, and other desired tests by following the assumptions of the software. By employing a bootstrapping approach, the study determined the level of significance (Hair et al., 2016).

### Ethics

#### Studies Involving Human Subjects and Obtaining Written Informed Consent

The ethics committee from the University and School of Sociology and Political Science reviewed, screened, and permitted this research project before the authors conducted the full study. The respondents recruited for this survey have provided written informed consent to the investigators and showed their willingness to participate in this study. The authors informed and educated the participants that they were conducting this survey according to the relevant University/institutional and national guidelines and rules, and that they were following the 's recommendations of the institutional/University ethics committee.

### Survey Duration: June 1 to November 30, 2018

The authors conducted this study from June 1, 2018 to November 30, 2018. The authors received informed written consent forms from the recruited human subjects involved in this study, which included their permission to publish the results. The investigators ensured that the research data were strictly confidential and did not disclose the identities of the participants to any other organizations or people. The authors provided permission to the study participants to interrupt the process of filling out the survey at any time if they had any concerns and needed any clarification. The principal investigators acknowledged that the respondents had cooperated in saving them time by filling out the forms for the survey.

### Preliminary List of Factors

Primarily, the investigators identified 18 influential factors affecting the perception of marital commitment of an individual. The authors conducted a pilot study to test normality, homogeneity, and independence statistics, and the pilot test provided satisfactory results to launch the primary investigation (Abbas et al., 2019a). Further, in the final step, this study involved 25 experts from the relevant field. It unified their endorsements to revise the self-reported study questionnaire by eliminating conflicting variables due to local conditions and the design of the proposed study. For factors affecting marital commitment, see Table 3.

**Table 3:** The identified factors list that influence marital commitment.

**Code**	**Influencing factors**
**Items**	**(A) Private religious practice (IV) (Lambert and Dollahite**, **2007****; Nelson et al.**, **2011****)**
PRP-1	I can feel God/Allah's presence
PRP-2	I can find strength in my spiritually or religion
PRP-3	I feel comforts/strength in my spiritually or religion
PRP-4	I can directly feel love of God for me
PRP-5	I am spiritually/religiously by the beauty of creation
PRP-6	During worship or other times, I feel joy that lifts me out when connect with God
**Code**	**Influencing factors**
**Items**	**(B) Religious support (IV) (Larson and Goltz**, **1989****; Lambert and Dollahite**, **2007****; Nelson et al.**, **2011****)**
RSF-1	How often do the people in your congregation make you feel loved and cared for?
RSF-2	How often do the people in your congregation listen to you talk about your private life?
RSF-3	How often do the people in your congregation express interest and concern in your well-being?
**Code**	**Influencing factors**
**Items**	**(C) Daily spirituality (IV) (Larson and Goltz**, **1989****; Lambert and Dollahite**, **2007****; Allgood et al.**, **2009****; Nelson et al.**, **2011****; Mosqueiro et al.**, **2020****)**
DSF-1	When you were a young child, how often did you attend religious services?
DSF-2	How often do you go to religious services?
DSF-3	Besides religious services, how often do you take part in other activities at a place of worship?
DSF-4	When you were a young child, how often did you participate in religious practices at home, either by yourself or with your family?
**Code**	**Influencing factors**
**Items**	**(D) Marital commitment (DV) (Lambert and Dollahite**, **2007****; Allgood et al.**, **2009****; Nelson et al.**, **2011****)**
MC-1	I am dedicated to making my marriage as fulfilling as it can be.
MC-2	A divorce would ruin my reputation.
MC-3	Marriages are supposed to last forever.
MC-4	When things go wrong in my marriage, I consider getting a divorce.
MC-5	I would not be embarrassed to get a divorce.

*This Table displays influencing factors on marital commitment in this study*.

### Designing the Questionnaire

This study applied a literature-based questionnaire to investigate the likely connotations between selected religiosity variables and marriage commitments of couples, and examined these linkages in the pilot study (Anjum et al., 2017; Hussain et al., 2021). On the recommendation of the experts, the investigators revised the study scale and distributed a modified version in combination with the selected elements of the self-reported scale among the target population to collect the required data from the participants. The study incorporated a random sampling technique to inform and train the study participants, and educate them about the purpose of the survey. The investigators assured the respondents that all data obtained would be strictly private and confidential. The selected questionnaire contained two parts to record feedback of the respondents on marital commitment. The first part of the self-reported instruments recorded the general information related to study respondents, for instance, their age, level of education, gender, residential areas, and profession/occupation. The second part of the instrument explained the impelling factors of religiosity that affect the marital commitment of people. This study recruited married couples from communities residing in five different regions of Pakistan and recorded their views about religious elements and their influence on the marital commitment of individuals. The selected instrument employs five points of the Likert scale to record the opinions of the participants. The investigators asked them to rate their extent of agreement on the scale ranging from the level of agreeing and strongly agree and disagree and disagree strongly (strongly agree = 5, neither agree nor disagree = 4, agree = 3, disagree = 2 and strongly disagree = 1).

### Statistical Sample Size Determined Using Cochran's Formula

Cochran's sample size formula helps researchers draw a sample size from the statistical population under study. According to assumptions of this formula, the sample size should be >5% of the overall statistical population (sample size >5 percentage of the total population) for categorical data at a 5% alpha value (error of 5%). The study has drawn the desired sample by incorporating the formula of Cochran at 95% confidence level with a 5% error margin, which was in accordance with selection inclusion criteria (Abbas et al., 2019b). The self-reported instrument consisted of two sections. The first part recorded the demographic information of the participants. The second part noted chosen variables with the Health and Safety Executive's (HSE) standard questionnaire. Cochran's formula helps draw desired sample size from the entire population required for the study under examination. The research design of the study is cross-sectional and used a stratified random sampling approach for the purpose of data collection (Bouza-Herrera, 2016; Abbas et al., 2020).

### Size of the Target Population, Sample Location, and Respondent Inclusion and Exclusion Criteria

The study recorded 508 valid responses from the recruited participants and collected desired data from the married couples residing in five different communities of Gilgit-Baltistan, a northern region of Pakistan. The study set the inclusion criteria as follows: rural respondents with a certain education level; for instance, at least a bachelor's degree. The researchers excluded uneducated participants from the survey and other respondents who did not provide feedback in the pilot test. They had not shown a clear interest in participating in the survey. The investigators educated and trained the survey respondents, informed them about the purpose, and requested them to return duly filled forms within 14 days.

### Data Processing of the Questionnaire Responses

The respondents provided the dully-filled instrument forms within the deadline. The investigators screened and scrutinized the received survey forms to confirm the accuracy of the received data. They identified 508 complete and valid responses of the study participants. This study employed the smart PLS software version 3.3.3 as an analytical tool to perform the analysis of the received data. The results of the statistical analysis provided valuable insights into the importance of religiosity factors. The study invited 15 experts of rural development studies who provided their expert opinion in the pilot test and requested them to provide their opinion on main study findings related to marital commitment. The results offered satisfactory relationships between the chosen variables of this model.

### Sampling Method and Population Size

This study incorporated the random sampling method to collect data from the targeted population. The authors subsequently checked and screened the collected data to verify their validity using the Smart PLS tool. This study integrated the sampling method to collect data from the targeted population of five regions in Pakistan, and the research focused on 508 respondents who provided complete responses. The participants in this study included males and females aged 18–60 years. This exclusive study focused on evaluating and examining religiosity and its dimensions to predict marital commitment among Pakistani couples. The researchers devised an inclusive strategy and a detailed literature review regarding marital commitment and religiosity affecting married couples. The first step was to formulate a theoretical understanding of the matter mentioned above. Therefore, the researchers conducted a comprehensive literature review focusing on books and papers published in high-quality journals. The secondary source for identifying marital commitment in this study showed the relationship between the variables selected from the correlation analysis. This study tested the chosen variables of marital commitment between couples. Table 3 shows the demographic characteristics of the respondents from the five-targeted urban areas by gender, age, education, and residential area.

### Descriptive Statistics

This study has employed a demographic questionnaire to record feedback of the participants in this study based on their age, residence location, and type of gender (Local Burden of Disease HIVC, 2021). The statistical sample of this research survey comprised 508 respondents with an equal proportion of 50%, including males (254/508) and females (254/508). The investigators examined the age factor between the range: (1) 20–24, (2) 25–29, (3) 30–34, and (4) > 35. The study findings specified that 37.58% of the age range of the participants was 20–24 years, 20.42% were in the age bracket of 25–29 years, and 21.73% were in the age group of 30–34 years. In comparison, 13.07% of the age range of the participants was over 35 years, respectively. The received datasets reported various fields, such as the education sector 30.06%, healthcare sector 28.43%, banking sector 22.54%, telecom sector 6.69%, and hotel industry 12.25%. See Table 4 below.

**Table 4:** Demographic statistics.

	**Respondents' gender**	
**Items**	**Number**	**Percentage**
Male	306	50%
Female	306	50%
**Respondents' age**		
20–24	230	37.58%
25–29	125	20.42%
30–34	133	21.73%
>35	80	13.07%
Over 40	44	7.18%
**Educational background of therespondents**		
Under metric	83	13.56%
Intermediate	181	29.57%
Graduated	261	42.64%
Master's	87	14.21%
**Respondents' job sector**		
Banking industry	138	22.54%
Education industry	184	30.06%
Health industry	174	28.43%
Hotel industry	75	12.25%
Telecom industry	41	6.69%

## Data Analysis

This segment summarizes the overall results of the various analyses executed on the empirical data obtained from the survey. PLS-SEM is a method used for evaluating structural equation models (Abbasi et al., 2021). In this study, the investigators employed the PLS-SEM technique to analyze the data collected during a survey in Pakistan. The foremost objective of this study was to find factors affecting marital commitment. The literature review helped identify 13 influencing factors related to religiosity and three factors of marital commitment. This study developed a conceptual framework to test the relationships of the elements of the proposed model. The study tested the established model by employing the Smart-PLS software version 3.3.3 (Ringle et al., 2018). The investigators measured the effects of religiosity factors (IVs) on marital commitment (DV) of married individuals, and classified the evaluation of the model into two methods to examine the relationships of the variables (Henseler et al., 2015). The study evaluated the first external dimension model and then assessed the second model, called the internal structure model (Kiani et al., 2018, 2021; Hosseini et al., 2019; Hashtarkhani et al., 2020). This study applied the Smart-PLS software to examine and justify the research model. See Table 5.

**Table 5:** Mean scores, standard deviation scores, skewness, and excess kurtosis values.

**Sr.**	**No.**	**Missing**	**Mean**	**SD**	**Excess Kurtosis**	**Skewness**
PRP_1	1	0	3.363	1.435	−1.283	−0.281
PRP_2	2	0	3.389	1.534	−1.343	−0.393
PRP_3	3	0	3.587	1.436	−1.196	−0.514
PRP_4	4	0	3.284	1.457	−1.429	−0.149
PRP_5	5	0	3.714	1.64	18.625	1.388
PRP_6	6	0	3.472	1.461	−1.296	−0.385
DSF_1	7	0	3.266	1.476	−1.358	−0.194
DSF_2	8	0	3.348	1.546	−1.443	−0.297
DSF_3	9	0	3.444	1.423	−1.288	−0.304
DSF_4	10	0	3.435	1.326	−1.071	−0.312
RSF_1	11	0	3.51	1.41	−1.231	−0.367
RSF_2	12	0	3.343	1.415	−1.298	−0.203
RSF_3	13	0	3.407	1.356	−1.154	−0.246
MCF_1	14	0	3.363	1.357	−1.198	−0.202
MCF_2	15	0	3.458	1.774	93.39	5.949
MCF_3	16	0	3.489	1.438	−1.174	−0.433
MCF_4	17	0	3.359	1.632	32.536	2.65
MCF_5	18	0	3.544	1.4	−1.112	−0.47

*SD, standard deviation; DSF, daily spiritual factors; MC, marital commitment; PRP, private religious practice; RSF, religious support factors*.

### Evaluation of Outer Measurement Model

The main objective of the outer measurement model was to gauge the reliability and validity of the examined variables (Kiani et al., 2017; Goshayeshi et al., 2018; Halimi et al., 2020; Hashtarkhani et al., 2020). The study calculated the internal consistency of the models by a reliability test of a single manifest and design (Bergquist et al., 2020; Pishgar et al., 2020; Azimi et al., 2021). On the other hand, the validity of the variables was verified based on centralized and discriminant validity (Henseler et al., 2009; Hair et al., 2017; Shabanikiya et al., 2020). A comparison shows that the reliability of a single patent variable with a latent variable by calculating the coherent external load higher than 0.7 was satisfactory (Henseler et al., 2009). Cronbach's alpha, composite reliability score (CR), and average variance extracted (AVE) are three PLS modeling tests that are useful in defining the converging rationality of measured structures (Shanmugapriya and Subramanian, 2015). The study measured a measurement model based on the aforementioned rules. For the outer-loadings results, see Table 6.

**Table 6:** Outer-loadings results.

**Codes**	**DS factors**	**MC factors**	**PRP factors**	**RS factors**
DSF_1	0.749			
DSF_2	0.803			
DSF_3	0.732			
DSF_4	0.712			
MCF_1		0.819		
MCF_2		0.702		
MCF_3		0.791		
MCF_4		0.714		
MCF_5		0.836		
PRP_2			0.704	
PRP_3			0.675	
PRP_4			0.767	
PRP_5			0.679	
PRP_6			0.699	
RSF_1				0.848
RSF_2				0.844
RSF_3				0.774
PRP_1				0.762

*DS, daily spiritual factors; MC, marital commitment; PRP, private religious practice; RS, religious support. See Table 7 that shows the results of validity and reliability of the study*.

**Figure 2:**
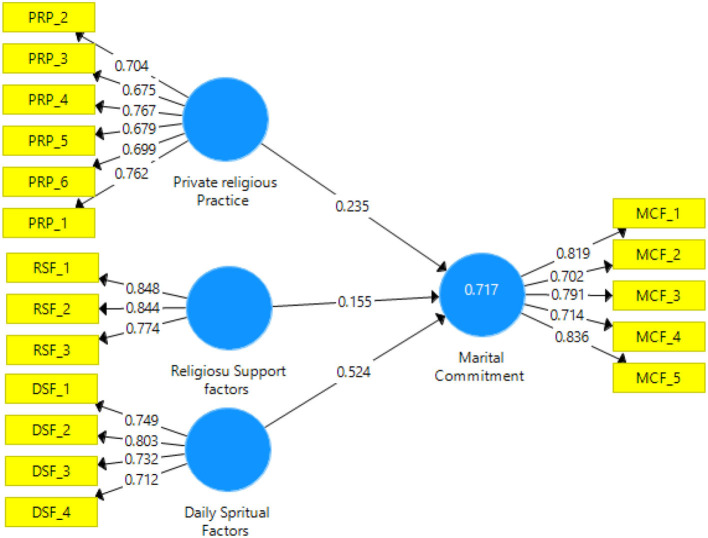
Algorithm model.

Table 7 presents the consistency and rationality of the results. The Cronbach's alpha and composite reliability values for the individual structures were higher than 0.7. The AVE values for all structures exceeded the critical 0.5 value. In addition, the loadings of all variables were within the permissible range, which was higher than 0.7. These findings show that the measurement model had adequate reliability and validity. See Figure 2 for the algorithm model and Tables 6, 7 below.

**Table 7:** Construct reliability and validity.

	**Cronbach's alpha**	**Composite reliability**	**AVE**
Daily spiritual factors	0.740	0.837	0.562
Marital commitment	0.831	0.882	0.599
Private religious practice	0.809	0.862	0.512
Religious support factors	0.760	0.862	0.677

*AVE, average variance extracted*.

### Evaluation of Inner Structural Model

The study applied the following method to gauge the structural model outcomes: noticing the prediction skills of the model and the relationship between the structures. The main way to measure an inner structural model is by the coefficient of determination (*R*^2^) of endogenous latent variables, which is essential for measuring an internal structural model. Other convenient methods for assessing an inner structural model are the path coefficient and t-statistics value, effect size (*f*^2^), goodness of fit, and predicted relevance of the model (*Q*^2^).

### Measuring the Value of R^2^

*R*^2^ is a measure of variance described by endogenous variables. Therefore, it can measure the predicted precision of a model (Sarstedt et al., 2014). The final model in this study had an *R*^2^ of 0.717, indicating that 71% of marital commitment differences were due to religious aspects. Falk and Miller (1992) suggested that *R*^2^ should be above 0.1 for a model to have projecting precision. If *R*^2^ is lower than that, the conceptual model is seen as incapable of defining the endogenous variables (Velayutham et al., 2012; Hirschi and Jaensch, 2015).

### Path Coefficients and T-Test Statistics

The path coefficient of the PLS model helps to achieve the standard beta coefficient (β) (Hair et al., 2017). The path coefficient indicates a potential change in endogenous variables to a single shift in exogenous variables. Compared with the conceptual or theoretical model using the β values of every trajectory, a higher β value has a more significant effect on the endogenous variables. The *t*-test is useful for calculating the significance of the path coefficients. The bootstrapping process helps in assessing the proposed hypothesis degree (Jiang et al., 2017). The significance of the value of the t-statistic must be equivalent to or higher than the limit value of 1.96 at 5% (Sarstedt et al., 2014; Hair et al., 2017). Table 8 presents the hypothesis testing results. See Table 8 below for the results of cross-loadings.

**Table 8:** Cross-loadings.

**CODES**	**DS**	**MC**	**PRP**	**RS**
DSF_1	0.749	0.631	0.515	0.606
DSF_2	0.803	0.685	0.613	0.575
DSF_3	0.732	0.589	0.619	0.643
DSF_4	0.712	0.555	0.523	0.557
MCF_1	0.713	0.819	0.624	0.613
MCF_2	0.591	0.702	0.480	0.492
MCF_3	0.626	0.791	0.569	0.556
MCF_4	0.570	0.714	0.491	0.504
MCF_5	0.677	0.836	0.665	0.643
PRP_2	0.580	0.560	0.704	0.471
PRP_3	0.552	0.534	0.675	0.482
PRP_4	0.630	0.559	0.767	0.559
PRP_5	0.434	0.457	0.679	0.478
PRP_6	0.432	0.477	0.699	0.487
RSF_1	0.647	0.578	0.569	0.848
RSF_2	0.624	0.590	0.529	0.844
RSF_3	0.676	0.627	0.583	0.774
PRP_1	0.590	0.559	0.762	0.458

**Figure 3:**
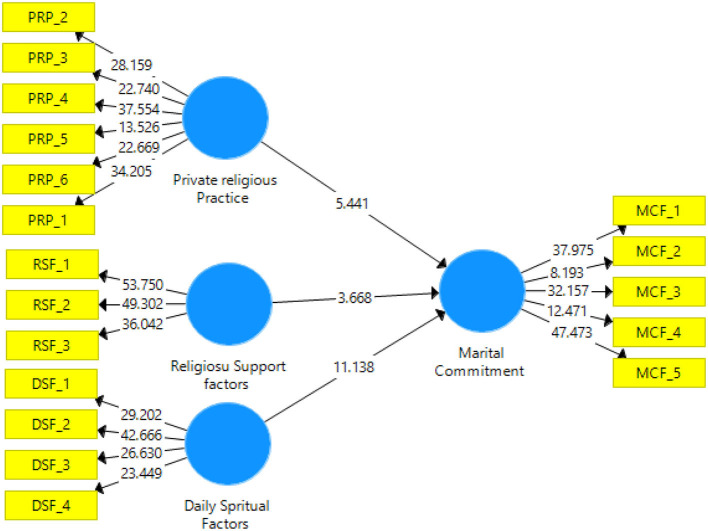
The *t*-test statistics.

The results stipulated in Table 9 concluded that the results supported the study hypotheses. Therefore, they established that the factors of religiosity considered in this study had a significant impact on marital commitment. See Figure 3 and Table 10 for the beta (β) and *t-*test statistics.

**Table 9:** Fornell–Larcker criterion.

**Factors**	**DS**	**MC**	**PRP**	**RS**
Daily spiritual factors	0.750			
Marital commitment	0.724	0.774		
Private religious practice	0.657	0.637	0.715	
Religious support factors	0.792	0.730	0.683	0.823

**Table 10:** Beta values (β) and t-statistic values.

**Hypothesized path**	**Standardized beta (β)**	***T*** **-test statistics**	***p*** **-value**
Private religious practice >MC	0.235	5.441	0.000
Religious support factors >MC	0.155	3.668	0.000
Daily Spirituals factors >MC	0.524	11.138	0.000

### Effect Size of the Model

According to Cohen (2016), the effect size is large if *f*^2^ ½ = 0.35, medium if *f*^2^ ¼ = 0.15, and small if *f*^2^ ¼ = 0.02 (Jiang et al., 2017). The effect size (*f*^2^) indicates the degree of the effect of the latent exogenous structure on the latent endogenous structures. Table 10 specifies the effect size of the model. Ultimately, the study results offered fair values. See Table 11 below for effect size.

**Table 11:** Effect size of the proposed study model.

**Exogenous latent variables**	**Effect size ***f***^2^**	**Total effect**
Private religious practice	0.076	Moderate
Religious support factors	0.030	Moderate
Daily spiritual factors	0.276	Moderate

### Predictive Relevance of the Model (*Q*^2^)

**Figure 4:**
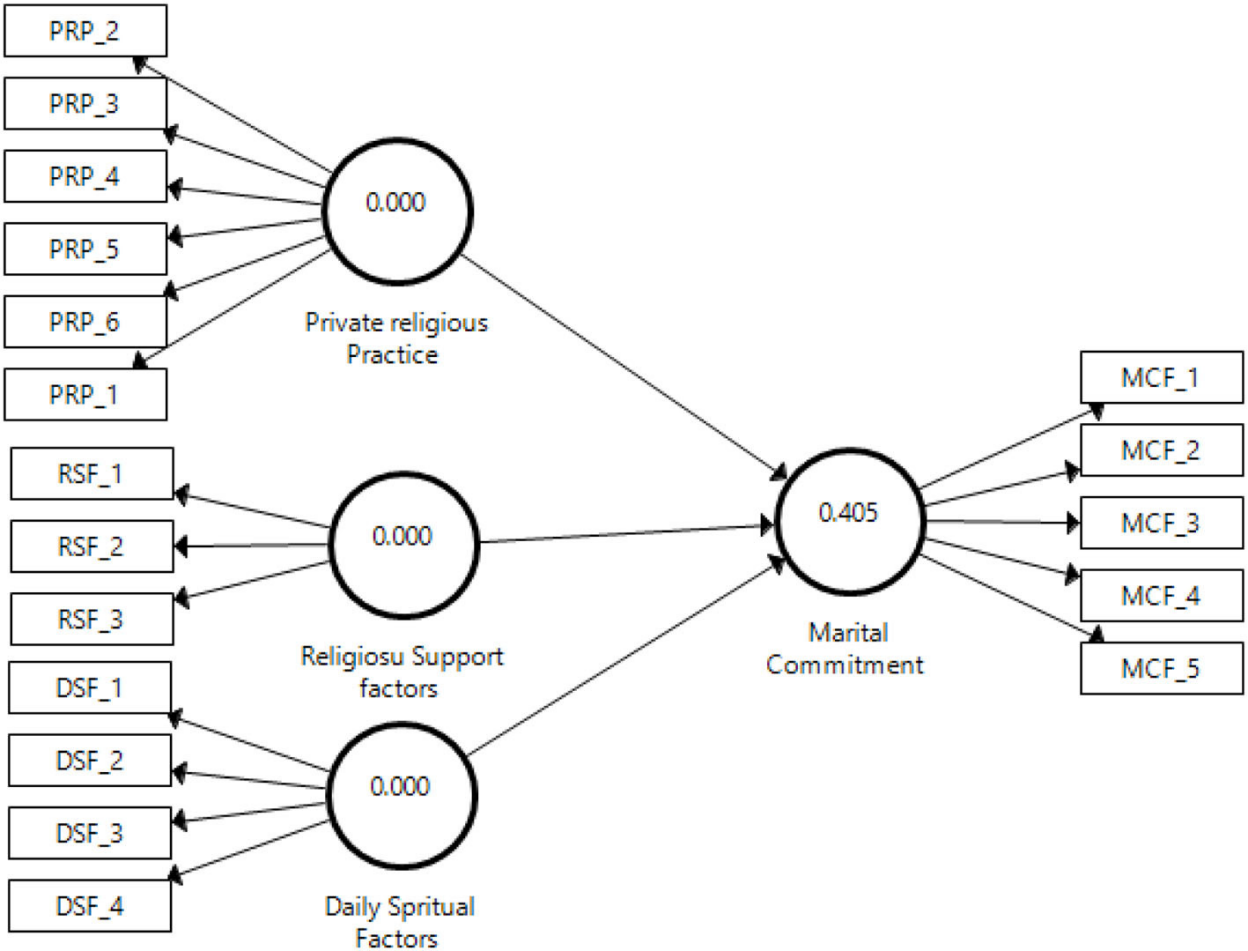
The blindfolding model.

This study applied the predictive relevance of the model -(*Q*^2^) by Stone Geisser for assessing the quality of the proposed model and the relevance of the model forecast. This model helps in computing the quality of the study model through the blindfolding method (Tenenhaus et al., 2005). In the blindfolding method, the *Q*^2^ standard assumes that values endorse the predicted significance of the model concerning a specific structure. A *Q*^2^ value higher than zero for the endogenous form indicates the projected relevance of the model for that paradigm, and lower *Q*^2^ values indicate predicted relevancy in the model (Sarstedt et al., 2014; Hair et al., 2017). The study results provided a clearer image of the influence of religious factors on marital commitment. They could assist in improving the set of relevant parameters and in using full information for a feasible relationship. Figure 4 shows that the *Q*^2^ value for this study model was 0.405, which was above the threshold limit, confirming that the predicted importance of the route model was significant for the endogenous structure. See Figure 4 for the blindfolding model (*Q*^2^).

## Discussion

This study primarily emphasized examining the function of religiosity factors and their influence on the marital commitment of individuals in the Pakistani community. The study findings have shown that the religious affiliation of individuals positively influenced marital commitment in the community of Pakistani married couples. Nevertheless, the empirical results of this study provided support to the links between religious factors (private religious practice, religious support, and daily spiritual factors) to strengthen marital commitment among married couples in Pakistan. Sociologists and anthropologists try to explain the critical significance of religious sociology and it focuses on the ways people live in society. Pakistani society thrives because of the consequences of theology, as 97% of its people are Muslims, and almost 2.5% are Christians. Therefore, the study results are helpful for decision-makers and policymakers in Pakistan, and the study findings provide valuable insights for other regions worldwide. The study focused on individual religious practices, beliefs, and societal manners, and how these factors influence the marital commitment of married people in Pakistan. The previous research findings have shown that Abrahamic religions (Islam and Christianity) share various identical values and similarities (Agius, 1998; Tremmel, 2009; Zarean and Barzegar, 2016; Agudelo and Cortes-Gómez, 2021; Glas and Spierings, 2021).

The branches of Abraham's religion promote and educate people to strengthen traditional family ties, advise people to get merry rather than cohabitate and keep marital relationships instead of non-marital births (Feldhaus and Heintz-Martin, 2015; Carlson and VanOrman, 2017; Koenig, 2018). Religions have some differences among people to practice religious beliefs. Christianity does not allow polygyny, whereas Islam permits it with certain conditions to practice justice among all wives, which might influence family model and marital commitment in some cases. However, in reality, there are rare cases of polygyny in Muslim communities in Pakistan and other societies worldwide (Al-Krenawi and Graham, 2006). Despite the differences in practicing religion, this study identified that religious affiliation promotes marital commitment and a healthier family model in Pakistan. The findings provide interesting insights for religious studies scholars to investigate other factors further to examine the religious influence on marital satisfaction among people. Western media highlighted family issues of the Muslim communities negatively in some cases. The problematic family issue, such as treating women and gender roles received importance in the society (Ennaji, 2016; Moufakkir, 2020; Chan and Tam, 2021). However, previous research has not supported unfavorable interpretations of situations of women, and religious Muslim couples showed prominent marital commitment and family satisfaction levels (Abdel-Khalek, 2006, 2010; Asamarai et al., 2008; Ahmadi and Hossein-abadi, 2009; Zaheri et al., 2016). Aman et al. (2019) described that marital commitment and satisfaction among Christian and Muslim spouses were different, and they showed relatively dissatisfaction; however, the religious affiliation of individuals had no effects on marital satisfaction (Aman et al., 2019).

The likely logic and elucidation support that people typically consider marriage a long-lasting relation (Silliman and Schumm, 2004; Willoughby and Dworkin, 2008). The marriage decision of a couple determines that they have rationalized and justified their cognitive choice, and closed other relationships (Webster and Kruglanski, 1994). The findings of this study specified that married couples from the Pakistani community reported good quality of marital commitment and religious affiliation has a positive influence on their quality of life together. The reason could be the couples feeling that they had invested a lot in this relationship development and better to maintain it. Internal differences and conflicts of couples might have surfaced. For instance, why am I keeping a relationship with her/him even this link makes me annoyed and unhappy? It needs to elucidate that the dissonance of maintaining unsuccessful relationships is harmful and may produce unpleasant emotions among people, particularly in a Western individualistic culture. This culture pursues personal happiness and well-being at all cost (Gilovich et al., 2015). Such personal emotions may also occur in all cultures and societies, such as Easter, a collective culture that highlights the importance of being grateful, selfless, and indebted to one's partner (Kagawa-Fox, 2010). The literature has documented mixed results, and the findings of this study are consistent with the literature and provide valuable insights from the collectivistic culture of Pakistan (Moradi et al., 2020; Shuja et al., 2020; Azadi et al., 2021). Social scientists examine how social aspects influence religion in a society, and the sociology of religion highlights communal and family lifestyles of people. The main objective of this study was to examine the connections between the recognized and selected variables and their relationship with marital commitment, as well as religiosity and its aspects, such as religious beliefs, rituals, emotional affection, and age differences among Muslim couples in five urban regions of Pakistan. This study analyzed the effects of religiosity and its components on marital satisfaction through linear and multiple regression analyses. Previous studies have shown that religiosity is closely associated with marital satisfaction among married people (Mahoney et al., 2001). In an Islamic society, a wedding is a religious ceremony in which couples affirm their allegiance to God. Religious-minded couples seem to have a more significant relationship with each other. Researchers conducted several studies on religion in Western countries to evaluate its influence and the relationship of religion with gender roles in a satisfying marriage. For instance, the survey of Zahra Algafli looked at observations of Islamic instructions and relations between married couples from an internal point of view. It reflected on essential matters, such as the fundamental rights of women, gender role classification, and family relations. Marital commitment and satisfaction are critical to creating a good marital relationship and a satisfactory marriage (Alghafli et al., 2014). Hence, this study aimed to assess the impacts of religiosity and other factors that influence the marital satisfaction of people to predict marriage quality between couples living in five different Pakistani cities. The study focused on educated and married urban respondents, and the results showed that religiosity and its aspects had a positive and significant correlation with marital commitment.

Religion explains the transformations in marital commitment, and its characteristics predict marital commitment among married inhabitants of cities. In addition, the results showed that the meaning of religious measurements showed maximum participation in the prediction of marital commitment. In contrast, rituals showed the lowest ability to predict marital commitment among couples. Hence, the results supported the stated hypothesis that religiosity and its characteristics can predict and strengthen marital commitment. The results of this study are in line with the findings of previous studies conducted by renowned scholars (Onyishi et al., 2012; Fard et al., 2013; Baussano et al., 2017; McDonald et al., 2017; Sorokowski et al., 2017; Mosqueiro et al., 2020). Therefore, commitment to marital capital also makes sense in terms of positive attachment aspects, since effective relationships between couples in marriages are critical. Eventually, religion encourages marital commitment, not only for the individual but also for the couple. Providing a holy goal for marriage and religion offers women the opportunity to fulfill their marital commitment.

The study results provide helpful insights. The findings are consistent with the existing literature, specifying that religiosity typically designated a more contented, stable, and healthier marital commitment among married people in Pakistan. The literature documents that religiosity develops intimacy and marital commitment among married people and supports marriage importance. It creates marital commitment among spousal relationships, which ultimately leads to happier and healthier marital quality.

Religious beliefs are foundational for husbands and wives, providing guidelines on sexual relations, gender responsibilities, and marriage-related sacrifices. If husband and wife have ideas about their future, marital commitment is eloquent for married couples' happy lives. Ultimately, religion is important to family life, since beliefs and religious connections are necessary when both spouses follow similar beliefs. Previous research has shown that religiosity acts as a social variable that controls the usual way of life of people. This study also revealed the controlling features of religion in married couples. Previous studies have demonstrated that religion plays a vital role in lowering the divorce rate, as it is a severe obstacle to divorce. In a broader sense, a couple ending their relationship in a society based around a religious community is very difficult, because there are many barriers to ending a married life. After all, religion supports people living together. However, non-religious couples face fewer obstacles, as there is an entire marital life for non-religious people (religion is a substantial barrier in this matter).

This study proposes another essential research direction for prospective studies to explore the role of spirituality and religiosity in global crises, such as the coronavirus disease 2019 (COVID-19) pandemic (Abbas, 2020, 2021; Yoosefi Lebni et al., 2020a,b; Su et al., 2021a). Future studies can investigate whether religious affiliation promotes marital commitment among married couples amid the COVID-19 pandemic. The studies can examine whether religious affiliation helps people amid the COVID-19 Crisis. Previous data about pandemics and infectious diseases can help expedite new research areas (Su et al., 2021b). This study raises concern about how religiosity helps people cope with challenging circumstances and remain satisfied in all complicated circumstances (NeJhaddadgar et al., 2020; Su et al., 2020; Maqsood et al., 2021). Past literature specifies that disease and virus outbreaks and pandemics pose mental health issues and create challenges for the entire humanity worldwide (Banerjee, 2020; Abbas et al., 2021a). There are numerous ways to reduce the adverse consequences of the pandemic, such as social media users interacting with friends and family through digital technologies, virtual working environments, and practices of religious beliefs (Galea et al., 2020; Koenig, 2020; Abbas et al., 2021b). The literature evidenced that various elements help promote and inhibit life satisfaction of individuals: a specific note of this study described that spirituality and religiosity play an indispensable role as religions help provide psychological resources, which have a positive linkage to psychological well-being (Barreto et al., 2015; Taufik et al., 2021; Wang et al., 2021). Internet use and innovative strategies amid COVID-19 pandemic provide updated information for better mental health solution (Jafari et al., 2010; Azizi et al., 2021; Khazaie et al., 2021). The scientific community reported that religion is one of the vital factors of the mental health individuals in the COVID-19 crisis, and religious beliefs provide mental relaxation to patients (Fardin, 2020).

Despite numerous studies on marital satisfaction (Bradbury et al., 2000; Twenge et al., 2003; Latha et al., 2011; Hilpert et al., 2016; Carranza Esteban et al., 2021), a large body of research studies has rarely controlled couples' religiosity factors, and these studies have not examined differences of various religious affiliations of the participants (Sullivan, 2001; Williams and Lawler, 2003; Olson et al., 2014). Future studies should pay attention to exploring the influence of religiosity affiliation of various religions and cultures and examine different factors that might affect marital commitment among people of various spirituality affiliations, education level, income, family size, and development level in the country. These indicators will help understand the interaction between religious beliefs, practices, marital commitment, and cultures of respondents (Zimmer et al., 2019). This study primarily emphasizes identifying the likely relations between marital commitment and religiosity factors among married people in Pakistan. The study results provide novel findings based on the context of a Pakistani sample of married people. The findings offer a valuable contribution to the literature designating religiosity effects on the marital commitment of married couples. Future studies can incorporate large sample sizes, different variables, and data sets from other regions to draw novel findings on this research direction.

## Conclusion

This study concluded that religious alignment has a profound influence on people and that it enhances religious commitment among couples by improving their awareness and determination to devote themselves to their families. The results showed that 173 (34.1%) individuals professed themselves very religious and that their marital commitment was firm because of their religious beliefs. In contrast, 255 respondents showed themselves to be religious and considered religion to be their priority. This result suggests that 428 respondents (84.3%) were very religious or religious, which indicates that Pakistani society has a strong link with religion and individuals are directly impacted by it. In contrast, 51 (10%) of the respondents declared that they were not religious, 23 (4.5%) couples did not practice any religious rituals, and 6 (1.2%) remained neutral in their beliefs. The findings also showed that religious beliefs were significantly able to predict (β = 0.764, *p* = 0) marital commitment. The results also demonstrated that religious rituals are of great importance in predicting marital commitment (β = 1.66, *p* = 0.025). Finally, the terms and conditions of marriage were significantly positively related to marital commitment (β = 0.58, *p* = 0.001). The hypotheses of this study were thus supported.

Previous studies examined whether belief in the God of people could help them by providing security, control, help, and advice. Married couples declare and recognize that they consider God in their lives, which gives them a more substantial marital commitment. This study demonstrated that religious people avoid improper extramarital relations, and their religious beliefs make them devoted to staying with their spouse only. In general, religiosity is the most influential factor that affects marital commitment between couples, helping to maintain their relationship. It is a source of family stability and ultimately ensures family satisfaction between couples. Therefore, the religiosity level of individuals is critical. Marriage is the first stage in the formation of a family, and religious people see it as a holy and divine notion recommended by God. People are drawn to marriage as a way to sustain their family center. Religious education also helps maintain marital commitments. It can be combined with religious education, focusing on beliefs to strengthen the religious-oriented practices of individuals and to increase the participation of couples in religious and social ritual meetings. This will promote marital relationships and develop marital satisfaction among couples, thus improving marital relationships. All of the influencing factors in this study, such as religiosity, beliefs, rituals, age differences, and marriage conditions, are essential for married couples. Still, religiosity is the most important and plays a significant role in maintaining married life among religious-minded couples.

### Limitations

This study focused on Muslim married couples from merely five selected cities in Pakistan, so the findings are not applicable to other regions and populations. Hence, further research studies should include substantial variability across diverse faiths to study marriage commitment and satisfaction among couples. The specific size of the sample cannot be considered as representing the whole of Pakistan. In addition, the research design did not allow causality to be deduced. A complete and inclusive evaluation of the respondents may overcome the deficiencies of the proposed survey. A qualitative research method or mixed research method is also recommended for future research. The authors eventually used just two aspects of religiousness, religious commitment and religious beliefs, during the execution of this study. Based on existing reviews, there are many other factors of religiosity that merit further investigation. Using a larger sample size to study other religiosity factors could provide more reliable results that could define the roles of these factors in marital commitment and ensuring a happy life among couples based in Pakistan.

Regarding limitations, this study also faced certain limitations related to the milieu of cultural and social norms and values of Pakistan, which have existed for a 1,000 years. Therefore, a fundamental limitation associated with this study was the strong influence of so-called religious scholars. This was a significant barrier to collecting survey data from female respondents, as such religious scholars influence them. These scholars strongly believe that women should not interact with men other than family members. The impact of this on rural inhabitants was considerably higher. Even male respondents were unwilling or hesitant to respond, which led to the exclusion of rural residents from this study during the pilot test. This study maintained the secrecy of the respondents and did not release any information that could reveal who the respondents were; individuals could even refrain from discussing their specific internal context.

The results showed that the responses did not necessarily reflect the respondents accurately. Some participants tried to present themselves as more religious than they were, while most of the respondents behaved well when responding to the survey. Further significant limitations related to this survey include the self-report tools used to complete this study.

### Suggestions

Finally, the study findings suggest that family therapists, family counselors, and married and experienced couples can play a pivotal or causative role in society by raising awareness of and commitment to marital satisfaction and high-quality relationships. This is possible through educating and encouraging other married people to maintain their marriages. They can inform others about the harmful results of divorce and its effects on families and children. On the other hand, marital commitment and marital satisfaction among married people can be improved/enriched through additional efficient and coherent measures based on the features of religious beliefs or thoughts by certain strategies that would support generalization and merit further study. Since there is insufficient research in the previous literature, various methods should be adopted to evaluate several maintenance strategies used by people who have had successful marriages.

Concerning the future aspects of such research studies, only few measures are essential for the evaluation. The validity of questions, any bias in the selection process, and desired research tools are crucial for absolute reliability. Therefore, these issues need proper consideration, and researchers need to solve the research problems before conducting their study. While it appears problematic, it still offers new opportunities. This will allow potential readers to gain a broader understanding of building a satisfied and devoted marital bond. Religiosity is an incentive for marital commitment and has a significant impact on married couples when they share common religious bonds and beliefs. Therefore, the results of this study suggest that religiosity features will be useful predictors for future studies. Additional studies could explore the relationship between religiosity, attachment style, and marital satisfaction given cultural values of Pakistani society.

## Data Availability Statement

The empirical data of this study is available upon the request from the corresponding authors.

## Author Contributions

JAman conceptualized the idea, contributed to study design and analysis, and completed the entire article, which included Introduction, Literature, Discussion, and Conclusion, and edited the original manuscript before submission. JAbbas conceptualized the idea, contributed to the study design, and wrote and edited the full manuscript, which included, Abstract, Introduction, Literature, Methods, Discussion, and Conclusion, and edited the original manuscript before submission. UL contributed actively to revise the manuscript to address the reviewer comments and reviewed and approved the final edited version of this research study. GS approved the final edited version and supervised this research study. All the authors contributed to this study and provided their consent to publish this study.

## Conflict of Interest

The authors declare that the research was conducted in the absence of any commercial or financial relationships that could be construed as a potential conflict of interest.

## Publisher's Note

All claims expressed in this article are solely those of the authors and do not necessarily represent those of their affiliated organizations, or those of the publisher, the editors and the reviewers. Any product that may be evaluated in this article, or claim that may be made by its manufacturer, is not guaranteed or endorsed by the publisher.
